# *Cornus officinalis* Extract Enriched with Ursolic Acid Ameliorates UVB-Induced Photoaging in *Caenorhabditis elegans*

**DOI:** 10.3390/molecules29122718

**Published:** 2024-06-07

**Authors:** Zengwang Yue, Han Liu, Manqiu Liu, Ning Wang, Lin Ye, Chaowan Guo, Bisheng Zheng

**Affiliations:** 1 School of Food Science and Engineering, South China University of Technology, Guangzhou 510641, China; yy1323834474@163.com (Z.Y.); m18384824390@163.com (M.L.); ye.lin@marubi.cn (L.Y.); 2 Research and Development Center, Guangdong Marubi Biotechnology Co., Ltd., Guangzhou 510700, China; liu.han@marubi.cn (H.L.); wang.ning@marubi.cn (N.W.)

**Keywords:** UVB, photoaging, *Cornus officinalis*, ursolic acid, *Caenorhabditis elegans*, SKN-1

## Abstract

Ultraviolet B (UVB) exposure can contribute to photoaging of skin. *Cornus officinalis* is rich in ursolic acid (UA), which is beneficial to the prevention of photoaging. Because UA is hardly soluble in water, the *Cornus officinalis* extract (COE) was obtained using water as the antisolvent to separate the components containing UA from the crude extract of *Cornus officinalis*. The effect of COE on UVB damage was assessed using *Caenorhabditis elegans*. The results showed that COE could increase the lifespan and enhance the antioxidant enzyme activity of *C. elegans* exposed to UVB while decreasing the reactive oxygen species (ROS) level. At the same time, COE upregulated the expression of antioxidant-related genes and promoted the migration of SKN-1 to the nucleus. Moreover, COE inhibited the expression of the *skn-1* downstream gene and the extension of the lifespan in *skn-1* mutants exposed to UVB, indicating that SKN-1 was required for COE to function. Our findings indicate that COE mainly ameliorates the oxidative stress caused by UVB in *C. elegans* via the SKN-1/Nrf2 pathway.

## 1. Introduction

Optical radiation is a new environmental health problem posed to human beings in addition to water, air, and noise pollution. Ultraviolet (UV) radiation from sunlight is considered to be the primary optical radiation that causes skin damage, a process that is referred to as photoaging [1]. Photoaging accounts for about 80% of skin aging [2]. UV radiation can be divided into short-wave, medium-wave, and long-wave ultraviolet radiation (UVC, UVB, and UVA, respectively) [3]. UVC is absorbed by the ozone layer, while UVA and UVB can reach the surface of the earth [4]. UVB photons are much more energetic than UVA and have a stronger cytotoxic effect [5]. UVB radiation can cause an excessive amount of reactive oxygen species, which can cause DNA damage and lipid peroxidation as well as protein modifications and degradation [6]. Studies have found that bioactive compounds from plants can prevent skin photoaging induced by UVB irradiation. For example, amla fruit extract could eliminate oxidative stress by reducing the production of intracellular ROS against UVB-induced keratinocyte inflammation and apoptosis [7]. Chrysin (5,7-dihydroxyflavone), a natural flavonoid, could attenuate apoptosis and ROS production induced by UVB [8]. Additionally, it was found that quercetin and its mixture (quercetin, luteolin and lycopene) prolonged the lifespan of UVB-irradiated *C. elegans* and reduced abnormal levels of ROS induced by UVB [9]. These studies are evidence that compounds in plants are candidates with great potential for use as active ingredients for ameliorating UVB-induced photoaging. 

*Cornus officinalis* is a medicinal and edible homologous plant that belongs to the *Cornaceae* family. As a traditional Chinese medicine, it is a famous health food and is extensively distributed throughout China. *Cornus officinalis* contains a variety of active ingredients, mainly including iridoid glycosides, polysaccharides, triterpenoids, phenols, and flavonoids, which grant its immune regulation, antioxidant, and anti-aging functions [10,11,12,13,14]. Thus, it is used for food and cosmetic purposes [15]. Among the triterpenoids in *Cornus officinalis*, ursolic acid (UA) is often evaluated in terms of relative content to assess the quality of *Cornus officinalis*. UA is a weakly polar pentacyclic triterpenic acid, which is a functional component of a variety of natural products and has significant antioxidant functions and other biological effects. A previous report has shown that UA, a dietary antioxidant, mediates its protective effect through the modulation of UVB-induced ROS in human lymphocytes [16]. In addition, UA could mitigate UVB-induced damage in human skin dermal fibroblasts by interfering with the ROS-mediated apoptotic induction and photoaging senescence [17]. However, the specific pathways through which UA mitigates UVB-induced damage in organisms have not been determined. Therefore, the *C. elegans* model was chosen to further investigate the effect of UA on UVB-induced photoaging in organisms. 

*C. elegans* is a popular model in aging research due to the simplicity of genes and the existence of homologous sequences with human genes in the genetic genome. Many signaling pathways related to aging in vivo have been identified. Moreover, *C. elegans* is also suitable for characterizing the damage caused by many stresses, such as UV irradiation, heat shock, and paraquat [18,19,20,21,22]. Studies have found that UV could induce an increase in ROS levels in *C. elegans*, resulting in a shortened lifespan, and that some plant components could alleviate ultraviolet stress to a certain extent. For instance, Tartary buckwheat extract could ameliorate the vital indicators of *C. elegans*, including the accumulation of ROS and malondialdehyde values caused by UVB exposure [23]. Ellagic acid could significantly increase the expression of SOD-3 and the activity of superoxide dismutase to clean out harmful ROS in *C. elegans* exposed to ultraviolet radiation stress [24]. Currently, a number of genes and pathways have been identified to regulate oxidative stress and prolong the lifespan in *C. elegans*, including the DAF-16/FOXO, SKN-1/Nrf2, and mitochondrial electron transport chain pathways. In *C. elegans*, SKN-1 is homologous to Nrf2 in mammals and plays an important role in defending against acute oxidative stress [25]. When *C. elegans* is exposed to acute oxidative stress, SKN-1 accumulates in the intestinal nuclei and activates phase II detoxification genes that correspond to canonical Nrf2 targets to directly control many critical detoxification processes [26]. Therefore, we considered whether oxidative stress in nematodes induced by UVB irradiation could be modulated by the SKN-1 signaling pathway. Based on the facts that *C. elegans* could be used as a model to study UV damage [27], and that UA could significantly lower ROS and prolong the lifespan of *C. elegans* [28], we studied the efficacy of the UA-containing components in *Cornus officinalis* against photoaging caused by UVB irradiation.

## 2. Results

### 2.1. Effects of the Crude Extract Diluted to Different Ethanol Concentrations on the Content of UA in the Supernatant and Redissolved Solution

**Figure 1:**
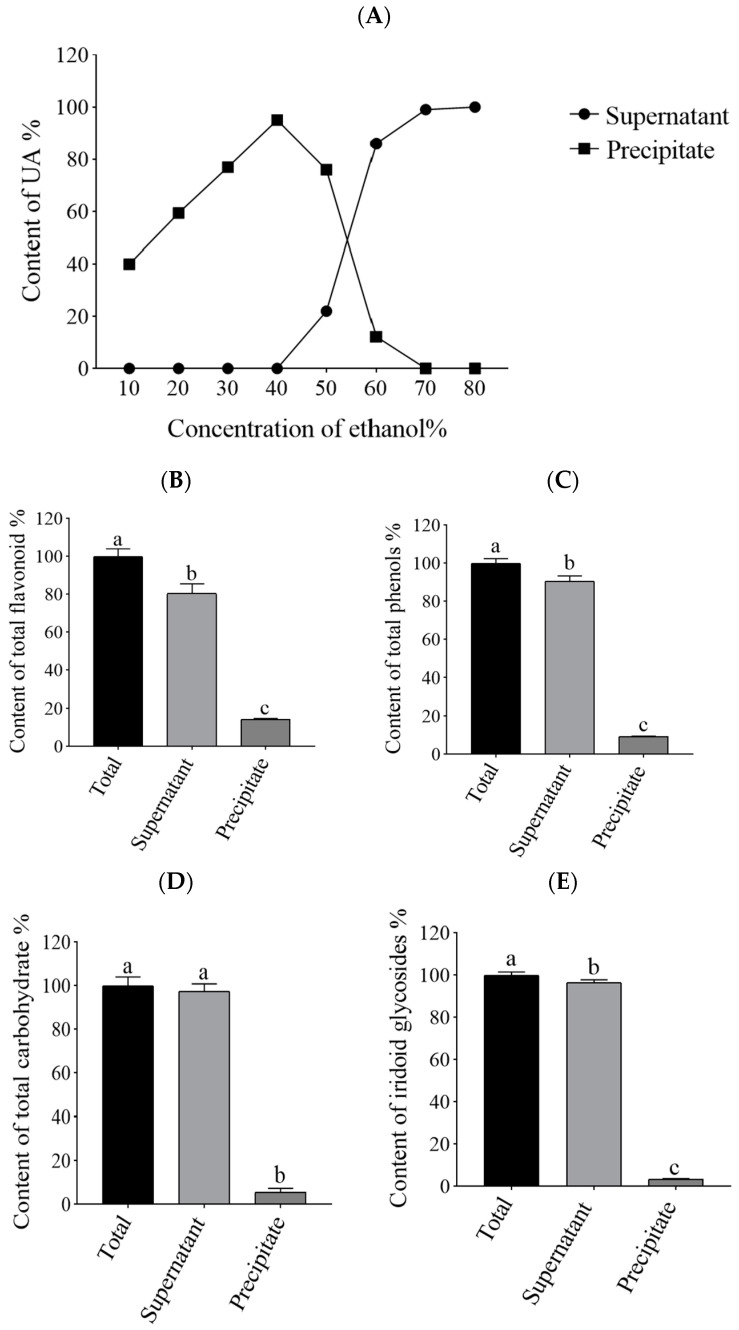
Distribution of various components in the supernatant and precipitate. (**A**) The proportion of UA content in the supernatant and precipitate when the crude extract of *Cornus officinalis* was diluted to different ethanol concentrations. (**B**) The proportion of total flavonoid content in the supernatant and precipitate when the crude extract of *Cornus officinalis* was diluted to 40% ethanol concentration. (**C**) The proportion of total phenols content in the supernatant and precipitate when the crude extract of *Cornus officinalis* was diluted to 40% ethanol concentration. (**D**) The proportion of total carbohydrate content in the supernatant and precipitate when the crude extract of *Cornus officinalis* was diluted to 40% ethanol concentration. (**E**) The proportion of total iridoid glycosides content in the supernatant and precipitate when the crude extract of *Cornus officinalis* was diluted to 40% ethanol concentration. Significantly different letters within each pairwise column indicate statistical significance (*p* < 0.05).

We added water to make UA precipitate as it is hardly soluble in water. The effect of ethanol concentrations in the range of 10–80% on the precipitation of UA was investigated. After dilution of the crude extract of *Cornus officinalis* to different ethanol concentrations, the UA content in the supernatant and redissolved solution was determined using high-performance liquid chromatography (HPLC). As shown in Figure 1A, the settled UA increased with decreased ethanol concentrations between 40 and 80% ethanol concentration. In addition, a decrease in supernatant UA content corresponded to an increase in redissolved solution UA content. Furthermore, it was observed that the maximum amount of UA was settled at an ethanol concentration of 40%. Decreases in ethanol concentration could increase the precipitation of UA. However, further decreases in ethanol concentration beyond 40% resulted in difficulty separating the UA pellet from the supernatant even when spin speed was increased or centrifugation time was extended. Part of the UA would neither completely precipitated nor completely be dissolved in the supernatant, resulting in filtration loss at the time of the assay, which may be due to the fact that the excess solution makes it difficult for UA to precipitate.

### 2.2. Content of Ursolic Acid

As shown in the Figure S1, we tested the content of UA in the samples. The specific results show that the content of UA increases from 21.75 µg/mg to 151.99 µg/mg after purification (Table S1). This method of preliminary purification has a good effect on improving the content of UA. 

### 2.3. Effects of the Crude Extract Diluted to 40% Ethanol Concentration on the Various Chemical Components

The components containing UA could be precipitated out by adjusting the ethanol concentration, and most of them could be settled at 40% ethanol concentration. We then proceeded to measure the changes in the content of the other components at a concentration of 40% ethanol to determine whether this concentration was beneficial for the separation of UA from other components. The results showed that the supernatant had significantly higher contents of phenols and flavonoids compared to the precipitate, and almost all iridoid glycosides and carbohydrate were distributed in it (Figure 1B–E). Thus, the ethanol concentration of 40% was chosen to make most of the other components transfer into the supernatant. As the majority of UA were distributed into the precipitate, it was convenient to separate the other components. Thus, we obtained this precipitate for subsequent experiments. 

### 2.4. Selection of UVB Dose

**Figure 2:**
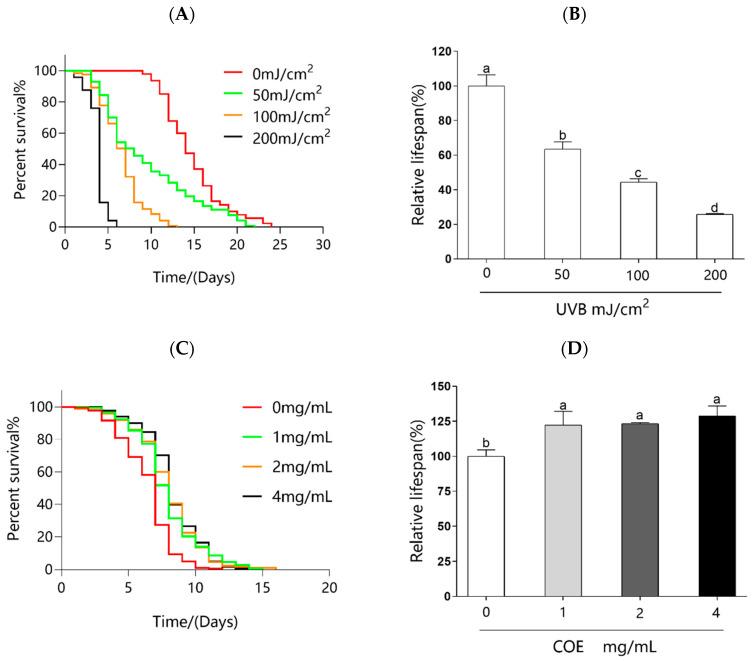
Effects of UVB doses on the lifespan of *C. elegans* and the effect of COE on the lifespan of *C. elegans* under UVB 100 mJ/cm^2^. (**A**) The lifespan of *C. elegans* under UVB irradiation, *n* > 70. (**B**) Relative lifespan of *C. elegans* exposed to UVB. (**C**) Effect of COE on the lifespan of *C. elegans* exposed to UVB 100 mJ/cm^2^. The treated nematodes were cultured with OP50 for 24 h and then exposed to UVB, *n* > 70. (**D**) Effect of COE on the relative lifespan of *C. elegans* exposed to UVB 100 mJ/cm^2^. A significant difference (*p* < 0.05) was observed between each column pair, as denoted by distinct letter labeling.

To investigate the optimal UVB dose, the worms were irradiated with UVB at doses of 0, 50, 100, and 200 mJ/cm^2^. The results show that the lifespan of the worms gradually decreased with the UVB irradiation dose from 50 to 200 mJ/cm^2^ (Figure 2A). Compared to the control group, the maximum lifespan of the group treated with 50 mJ/cm^2^ UVB did not significantly change (Figure 2B). The maximum dose of 200 mJ/cm^2^ did injure the worms and led to a 70% decrease in mean lifespan (Figure 2B), which made it difficult to observe the effect of COE. The irradiation dose of 100 mJ/cm^2^ significantly reduced the mean lifespan of *C. elegans* by around 50% (Figure 2B). This was determined to be the favorable dose for the outcomes of the study. Therefore, a UVB dose of 100 mJ/cm^2^ was selected for further experiments.

### 2.5. Effects of COE on the Lifespan of C. elegans after UVB Radiation

To determine the effect of COE on photoaging, the worms were cultured on NGM plates containing 0, 1, 2, and 4 mg/mL COE, and their lifespan was measured after being exposed to UVB radiation. Figure 2C,D show the effect of COE on the lifespan of *C. elegans* under UVB radiation. Compared to the COE-untreated nematodes, 1, 2, and 4 mg/mL COE significantly prolongs the lifespan of the nematodes by 22.39%, 23.33%, and 28.77%, respectively. The lifespan assays of nematodes in the COE-treated groups were significantly longer after exposure to UVB, indicating that the COE had a significant UVB protective effect on the nematodes. However, COE protected *C. elegans* from UVB-induced damage in a dose-independent manner.

### 2.6. Effects of COE on the Accumulation of ROS

**Figure 3:**
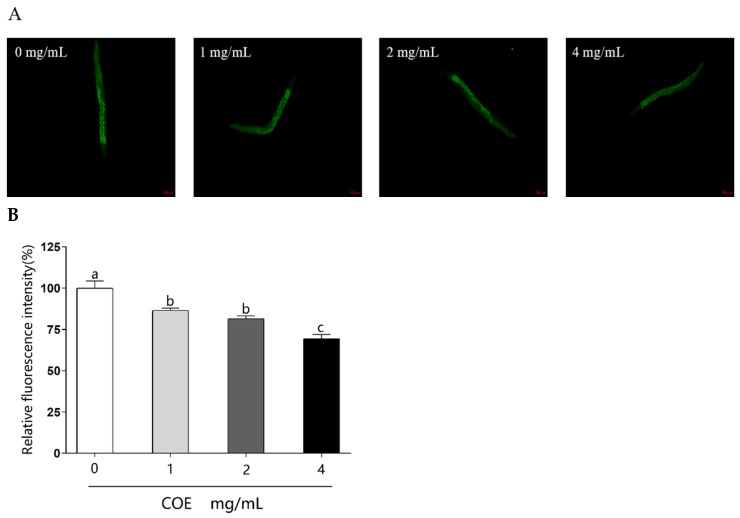
Effect of COE on ROS accumulation in *C. elegans* exposed to UVB. The treated nematodes were cultured with OP50 for 24 h and then exposed to UVB, *n* > 30. (**A**) Intracellular ROS in each group. (**B**) Quantification of the fluorescence intensity of ROS by Image J software 8.0. Values with different letters in each bar are contrasting (*p* < 0.05) significantly.

UVB radiation could cause a large accumulation of ROS [7]. Overexpression of ROS levels could activate signaling pathways that result in a series of oxidative stress reactions, which finally leads to skin photodamage [17]. Therefore, exploring strategies for decreasing the levels of ROS is an important area of anti-photoaging. The 2, 7-dichlorodihydrofluorescein diacetate (DCFH-DA) reagent was used to detect the ROS levels in nematodes exposed to UVB radiation stress. As shown in Figure 3, ROS level, indicated by fluorescence intensity, was significantly decreased in a dose-dependent manner in the worms treated with COE. Compared to the untreated group, the ROS levels in the treatment groups of COE were reduced to 92.27%, 81.57%, and 69.44%, respectively. The results indicate that COE could significantly reduce the overexpression of ROS caused by UVB.

### 2.7. Effects of COE on the Activities of Antioxidant Enzyme

**Figure 4:**
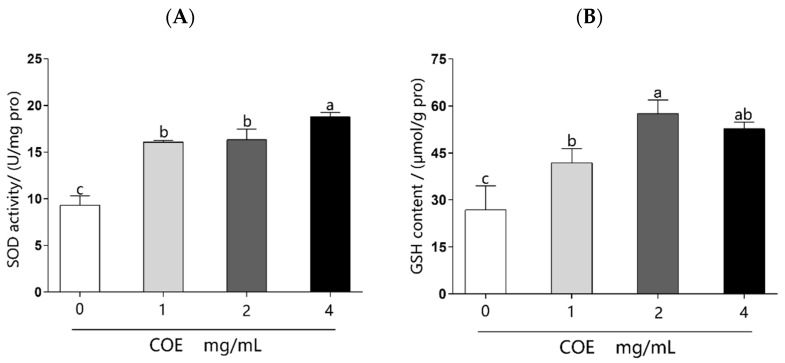
COE on the antioxidant enzyme activities in *C. elegans* exposed to UVB. The treated nematodes were cultured with OP50 for 24 h and then exposed to UVB, *n* > 1000. (**A**) SOD activity. (**B**) GSH content. Values with different letters in column for each figure are significantly different (*p* < 0.05).

The activities of antioxidant enzymes SOD and GSH are important indicators of oxidative stress. Related assay kits were used to determine the antioxidant enzyme activities in the worms exposed to UVB radiation. As illustrated in Figure 4, in the treatment group of COE, SOD activity was significantly improved by 0.72–1.01 fold, and GSH content was obviously enhanced by 0.56–1.14 fold compared to the control group. These results illustrate that treatment with COE significantly improves antioxidant enzyme activity in *C. elegans* after exposure to UVB radiation.

### 2.8. Effects of COE on the Expression of Oxidative Stress-Related Gene

**Figure 5:**
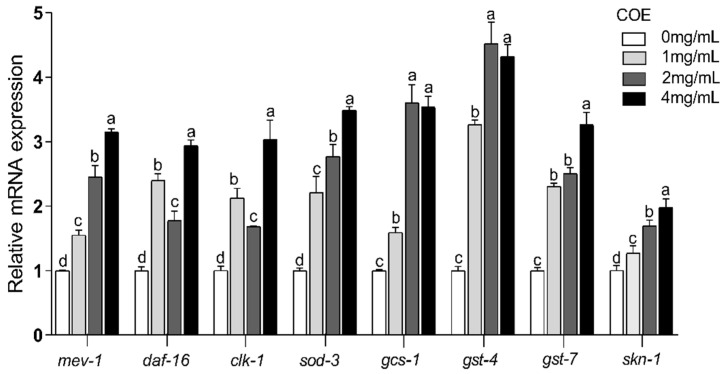
Effect of COE on oxidative stress-related genes expression in *C. elegans* exposed to UVB. The treated nematodes were cultured with OP50 for 24 h and then exposed to UVB, *n* > 1000. Bars of the same gene with different letters are significantly different (*p* < 0.05).

The results above indicate that COE effectively mediates the extension of the lifespan of *C. elegans* exposed to UVB radiation. To unravel the mechanism behind this effect at the transcription level, we need to measure the relative expression levels of oxidative stress-related genes through quantitative real-time polymerase chain reaction (q-RT-PCR). As displayed in Figure 5, the expression of the *mev-1*, *daf-16*, *clk-1*, *sod-3*, *gcs-1*, *gst-4*, *gst-7*, and *skn-1* genes were increased to 3.15 ± 0.05, 2.94 ± 0.09, 3.03 ± 0.30, 3.49 ± 0.05, 3.60 ± 0.27, 4.52± 0.34, 3.26 ± 0.20, and 1.98 ± 0.13, respectively, after the treatment with COE (*p* < 0.05). These results demonstrate that COE can relieve UVB mediated photoaging damage by regulating the expression of antioxidant-related genes.

### 2.9. Effects of COE on the Lifespan of skn-1(zu135) Mutants

**Figure 6:**
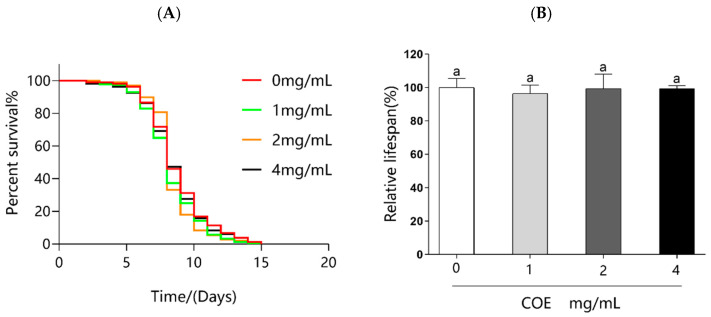
Effect of COE on the lifespan of *skn-1(zu135)* mutants exposed to UVB. The treated nematodes were cultured with OP50 for 24 h and then exposed to UVB, *n* > 70. (**A**) Percentage survival of nematodes. (**B**) Relative lifespan of nematodes. A significant difference (*p* < 0.05) was observed between each column pair, as denoted by distinct letter labeling.

Indeed, our results reveal that COE not only exerts direct antioxidant effects but also significantly upregulates the expression of *skn-1* gene. Therefore, we used *skn-1* loss-of-function mutant worms to investigate if the effects of COE on *C. elegans* acted through the *skn-1* gene. As shown in Figure 6 and Table 1, no lifespan extension is observed in the *skn-1* mutants, thereby strongly indicating that the *skn-1* gene is required for the COE mediated extension of lifespan in *C. elegans* after being exposed to UVB radiation.

### 2.10. SKN-1 Nucleus Localization

**Figure 7:**
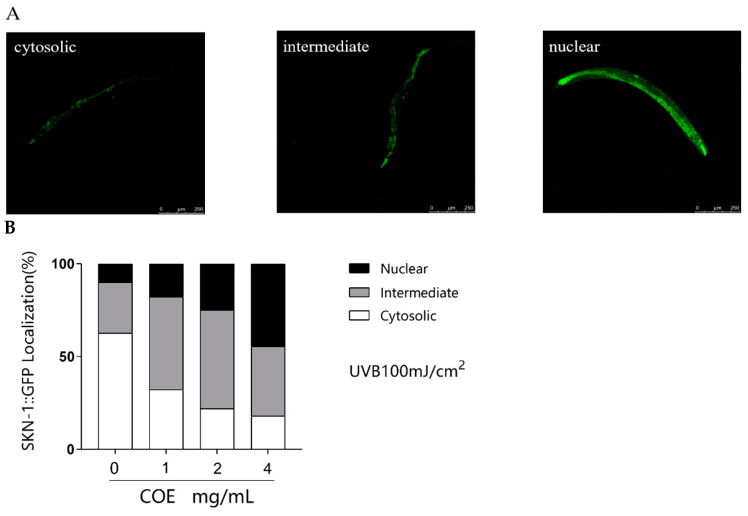
The effect of COE on the SKN-1::GFP translocation in the worms exposed to UVB. The treated nematodes were cultured with OP50 for 24 h and then exposed to UVB, *n* > 30. (**A**) Representative photos of SKN-1::GFP transgenic worms with cytosolic, intermediate, and nuclear SKN-1::GFP localizations. (**B**) The ratio of worms in each category.

In order to test the impact of COE on the intracellular localization of SKN-1 under oxidative stress caused by UVB, we employed a transgenic *C. elegans* strain stably expressing a SKN-1::GFP fusion protein. GFP was used to reflect this translocation in the present study. As shown in Figure 7A,B, COE treatment induces a reduction in cytosolic SKN-1::GFP fraction from 62.50% to 17.85% and an increase in nuclear SKN-1::GFP translocation from 10.00% to 44.64%. This phenomenon shows that COE treatment can affect the subcellular distribution of SKN-1 and induce a partial translocation of SKN-1 to the nucleus from the cytoplasm after being exposed to UVB radiation.

### 2.11. Effects of COE on the Expression of the skn-1 Downstream Gene

**Figure 8:**
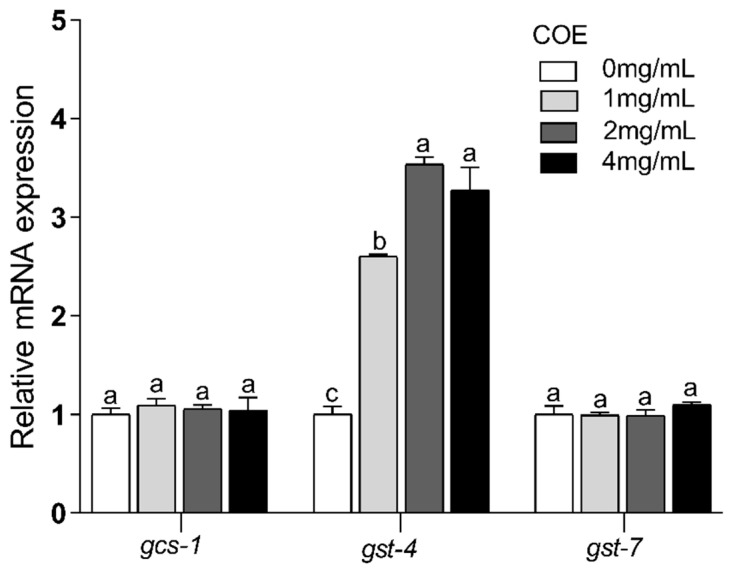
Relative expression levels of *skn-1* downstream genes in *skn-1(zu135)* mutants exposed to UVB. The treated nematodes were cultured with OP50 for 24 h and then exposed to UVB, *n* > 1000. Bars of the same gene with different letters are significantly different (*p* < 0.05).

The above results suggest that oxidative stress is closely related to the regulation of SKN-1 and that transcription factor SKN-1 could regulate downstream genes to modulate the oxidative stress of *C. elegans* caused by UVB. Therefore, we examined the expression levels of downstream genes of *skn-1* (*gcs-1*, *gst-4*, and *gst-7*) to explore the underlying anti-photoaging mechanisms of COE further. These results indicate that treatment of COE can significantly regulate the expression of *gcs-1*, *gst-4*, and *gst-7* genes in *C. elegans* exposed to UVB radiation (Figure 5). The gene expression of the COE-untreated group is defined as one. In the COE-treated group, the relative expression levels of *gcs-1*, *gst-4*, and *gst-7* are upregulated to 3.60 ± 0.27, 4.52 ± 0.34, and 3.26 ± 0.20 folds, respectively. However, in the *skn-1(zu135)* mutants exposed to UVB radiation, the COE-treated groups do not show significant differences in the expression of *gcs-1* and *gst-7*; the expression of *gst-4* is slightly increased (Figure 8). However, compared with the changes in N2 nematodes, the upregulation of the *gst-4* gene in the *skn-1(zu135)* mutant strain is significantly inhibited.

## 3. Discussion

The skin, which is the outermost organ and the first defense system of the human body, has the function of resisting external stimulation and exogenous hazards [3]. UVB is the most important factor that causes skin photoaging. Thus, inhibition of UVB-induced photoaging of the skin provides a rational basis for the development of cosmetic products and for the treatment of diseases caused by UV radiation [29]. Studies have indicated that plant extracts have dermato-protective properties against UVB damage. For example, Perilla frutescens extracts were found to enhance DNA repair in skin and keratinocytes exposed to UVB [30]. Previous studies have demonstrated the effects of *Cornus officinalis* ethanol extracts. For instance, ethanolic extracts of *Cornus officinalis* exert inhibitory effects on oxidative stress, allergic responses, and inflammatory responses [31]. In addition, they also show antioxidant effects against PM2.5-induced oxidative stress [32]. Simultaneously, *Cornus officinalis* is rich in UA, which has anti-photoaging effects [16,17]. However, the anti-photoaging activity of *Cornus officinalis* ethanol extracts based on UA has not been reported yet. Because UA is hardly soluble in water, we came up with a solution in which the partial water-soluble impurities (such as phenols, flavonoids, iridoid glycosides, and carbohydrates) are transferred to the supernatant in the preliminary purification step. Then, the effect of different ethanol concentrations on the precipitate of UA is investigated by adding certain volumes of water to the crude extract. The results show that an ethanol concentration of 40% precipitated the UA out, while the vast majority of the other components were left in the solution. Similar research also used water as the antisolvent for the recovery of UA from the crude extract of *Cynomorium songaricum Rupr* [33]. This impurity removal has the advantages of low cost, simple operation, and good environmental compatibility. In this study, a component containing UA was preliminarily purified from the crude extract of *Cornus officinalis* using water as the antisolvent, which we called the *Cornus officinalis* extract. Our study revealed that COE could prolong lifespan and ameliorate oxidative stress in *C. elegans* after UVB irradiation. Our finding of its anti-photoaging effect contributes to broadening the knowledge regarding the applied biology of *Cornus officinalis*, such as in producing new cosmetic ingredients. 

It is well known that UVB radiation can result in oxidative stress [7]. However, enhancing the activity of antioxidant enzymes would result in the scavenging of excessive free radicals in the body and would reduce oxidative stress [34]. It was found that COE could enhance the activity of antioxidant enzymes and decrease ROS levels, showing a remarkable inhibition of UVB-induced oxidative stress. In *C. elegans*, the signaling pathways involved in oxidative stress include the DAF-16/FOXO, mitochondrial respiration, and SKN-1/Nrf2 pathways. DAF-16 is a fundamental component of the DAF-16/FOXO pathway that influences the expression of many genes and their active protein products [35]. As the downstream target genes of *daf-16*, *sod-3*, and *gst-4* are essential for oxidative stress [36], results showed that COE significantly increased the expression of *daf-16* as well as its downstream *sod-3* and *gst-4* genes to increase the stress resistance of *C. elegans*. It has been reported that *clk-1* and *mev-1* genes in the mitochondrial respiration pathway play an important role in regulating lifespan and oxidative stress tolerance [37]. Our findings show that COE upregulates the expression of *mev-1* and *clk-1* to decrease the damage of UVB irradiation on *C. elegans*. 

In vertebrates, Nrf2 is responsible for inducing the expression of genes required for detoxifying stress and promoting longevity [38]. Nrf2 is evolutionarily conserved and present in *C. elegans*, where it is known as SKN-1. SKN-1 functions similarly to Nrf2 proteins in responses to oxidative stress and is required for oxidative stress resistance and longevity. In the intestine, the main detoxification organ in *C. elegans*, SKN-1 accumulates in the nuclei and activates target genes in response to environmental stresses. Activated SKN-1 could upregulate the expression of genes including glutathione S-transferase 4 (*gst-4*), the g-glutamine cysteine synthetase heavy chain (*gcs-1*), glutathione S-transferase 7 (*gst-7*) [39]. Therefore, the SKN-1/Nrf2 signaling pathway, which plays a crucial role in stress resistance and lifespan regulation in *C. elegans*, was mainly analyzed in our study. Our findings reveal that treatments with COE cannot extend the lifespans of *skn-1* mutant worms after UVB radiation, suggesting that the longevity effect of COE was mediated through the activity of the SKN-1/Nrf2 signaling pathway. We further investigated the effects of COE on the expressions of SKN-1 by GFP labeling and the target genes of the transcription factor SKN-1 in *skn-1* mutant worms. Treatment with COE significantly induced SKN-1::GFP nuclear translocation, in which SKN-1 gradually relocated from the cytoplasm to the nucleus to regulate the expression of downstream *gcs-1*, *gst-4*, and *gst-7* genes in response to oxidative stress caused by UVB. Interestingly, in *skn-1(zu135)* mutants exposed to UVB radiation, the relative expression levels of *gst-4* were still upregulated. Several studies have indicated that *gst-4* is a downstream gene in the DAF-16 pathway [40,41]. Therefore, we infer that the inconsistent behavior of *gst-4* gene expression in this experiment may still be influenced by the DAF-16 pathway, which is consistent with the increased expression of the *daf-16* gene above. These results indicate that COE could regulate the *skn-1* downstream gene by activating the transcription factor SKN-1 to promote expression of downstream genes and then prolong the lifespan of *C. elegans* exposed to UVB radiation. Taken together, COE activates the SKN-1/Nrf2 signaling pathway for lifespan extension and oxidative stress resistance in *C. elegans* exposed to UVB and that SKN-1 is necessary for COE to extend lifespan, which is similar to the findings of other studies [42]. 

Collectively, this study found that COE could increase the resistance of *C. elegans* to UVB stress. According to our findings, it is likely that the effect of COE ameliorating UVB damage involves the DAF-16/FOXO, mitochondrial respiration, and SKN-1/Nrf2 pathways. Additionally, the study indicates that activating SKN-1 is required for COE-mediated UVB stress resistance in *C. elegans*. This is of great significance for the development of protective agents that increase UVB resistance by using *C. elegans* as an animal model in the future. More experiments, such as transcriptomics, proteomics, and related mutant assays, must be conducted to further explore the specific regulation pathway of COE against UVB damage.

## 4. Materials and Methods

### 4.1. Preparation of Cornus officinalis Extract

Dried fruits of *Cornus officinalis* were obtained from local markets in China. UA were extracted from the sample using ultrasonic-assisted ethanol [43], and the major factors influencing the extraction were optimized to obtain the optimal extraction conditions. The crude extract of *Cornus officinalis* was obtained by extracting it for 40 min in 80% ethanol at a solid–liquid ratio of 1 g:15 mL and with an ultrasonic power of 100 W. UA is insoluble in water. Thus, when the crude extract was diluted to different ethanol concentrations by adding water, a precipitate was formed. The precipitate was redissolved in 80% ethanol after centrifugation and filtration. The content of UA and other components in the supernatant and the redissolved solution was determined. The COE was obtained by diluting the crude extract to the required ethanol concentration and centrifugation, followed by filtration and drying. The COE was used for subsequent research on the anti-photoaging effect on nematodes.

### 4.2. Determination of Chemical Composition

The phenol content was determined using a modified Folin–Ciocalteu colorimetric method reported in a previous study, and gallic acid was used as a standard [44]. The carbohydrate content was measured by the phenol–sulfuric acid method with D-glucose as the standard [45]. The flavonoid content was measured using the sodium borohydride/chloranil method using catechin hydrate as a standard [46]. UA content was quantified by HPLC, using pure standards as reference for retention time and quantification [47]. The main iridoid glycoside component of the extract was loganin. The loganin standard was scanned at 200–500 nm at the full wavelength of UV to determine its maximum absorption peak wavelength, which was found to be 237 nm. Thus, the total iridoid glycoside content was measured at the absorbance value of 237 nm [48].

### 4.3. Caenorhabditis elegans Strains

The wild-type *C. elegans* strain N2 (Bristol) and mutants, including *skn-1(zu135) IV* and LD1:ldIs7[*skn-1* b/c::GFP *rol-6 (su100)*] used in this study were obtained from the Caenorhabditis Genetics Center (CGC). The eggs were incubated on nematode growth medium (NGM) agar plates at 20 °C, seeded with live *Escherichia coli* OP50 (*E. coli* OP50) as a food source. COE treatment started from the first day of hatching. Unless expressly stated otherwise, the treated worms were transferred onto corresponding new plates every day during the propagation period, with added 5-fluoro-2-deoxyuridine (FUDR) to inhibit the spawning of worms. COE was dissolved in dimethyl sulfoxide (DMSO), then diluted to different concentrations of COE with *E. coli* OP50 (final concentrations: 1, 2, 4 mg/mL). For the group without COE, DMSO was added for the control group to make the final concentration of DMSO in the different groups the same.

### 4.4. UVB Irradiation Procedure

UVB radiation was carried out using a Spectrolinker CL-3000M UVP Crosslinker (Analytik Jena, Upland, CA, USA), which emitted the wavelength of UVB radiation at 302 nm. The output of UVB irradiation dose and efficiency from the lamp were measured using a radiometer. The *C. elegans* samples were exposed to various intensities of UVB (50, 100, 200 mJ/cm^2^). Control samples were processed identically but without UVB radiation. The UVB source was positioned above the NGM plates during the irradiation period. After being exposed to UVB radiation, the worms were transferred onto new plates with a thin layer of *E. coli* OP50.

### 4.5. Lifespan Assay

The determination of the lifespan of *C. elegans* was conducted according to a slightly modified method previously described [20]. *C. elegans* were cultured on NGM plates seeded with *E. coli* OP50 at 20 °C until the spawning period. Then, synchronous nematodes were obtained using a bleaching buffer treatment. COE treatment started from the first day of hatching. During the propagation period, *C. elegans* were transferred to NGM plates containing different concentrations of extracts and FUDR every day to prevent spawning. After the propagation period, *C. elegans* were transferred to NGM plates for UVB irradiation. After being exposed to UVB radiation, the worms were transferred onto new plates with a thin layer of *E. coli* OP50. Worms were scored daily by gently prodding them with a platinum wire, and abnormal worms were excluded. Statistical analyses were performed using the Graphpad Prism 8.0.2 software, and all assays were carried out in triplicate.

### 4.6. Measurement of ROS

ROS levels were determined using 2, 7-dichlorodihydrofluorescein diacetate (DCFH-DA) as previously described with some modification [9]. The treated nematodes were collected and loaded with 100 µM DCFH-DA (Sigma, St Louis, MO, USA) dissolved in M9 buffer for 0.5 h in the dark. Next, the nematodes were washed with M9 buffer three times and then placed on 2% (*w*/*v*) agarose pads on glass slides and anesthetized with sodium azide (10 mM). The worms were visualized under a fluorescence microscope (MF54-N, Mshot, Guangzhou, China). The fluorescence intensity of the worms was measured using Image J software 8.0 (National Institutes of Health, Bethesda, MD, USA).

### 4.7. Assay of Antioxidant Enzyme Activity 

Combined with the method reported in a previous study [24], the treated nematodes were collected in M9 buffer after UVB irradiation, washed three times, resuspended in M9 buffer, and then crushed into a homogenate with a biological grinder on ice. After centrifugation, the supernatant was collected for experimentation. The activities of antioxidant enzymes SOD and GSH were detected with the corresponding assay kits following the manufacturer’s instructions. Final data were calibrated to protein levels using BCA protein assay kit. The experiment was conducted in three independent trials with at least 500 nematodes in each group.

### 4.8. Quantitative Real-Time Polymerase Chain Reaction

Total RNA was isolated from around 1000 worms using TRIzol reagent (Invitrogen, Carlsbad, CA, USA) after UVB irradiation [49]. Then, these total RNA were synthesized into cDNA using the PrimeScript^TM^ RT Reagent Kit (Takara Biotechnology, Dalian, China). Subsequently, q-RT-PCR was conducted using the BioRad Mini Option^TM^ Real-Time PCR Detection System (BioRad, Hercules, CA, USA) with the SYBR Green Supermix fluorescence dye. The expression levels of genes were analyzed using the 2^−∆∆Ct^ method, and *actin* was chosen as the reference gene. The primers used for q-RT-PCR are as follows.


**Gene Name**


**Primer Sequences**

*Caenorhabditis elegans actin*
FGCTGGACGTGATCTTACTGATTACC 
RGTAGCAGAGCTTCTCCTTGATGTC
*Caenorhabditis elegans mev-1*
FGCCCAATCGCTCCACATCTCAC
RGAGAAGGGTTCCGGCCATTAC
*Caenorhabditis elegans daf-16*
FCGTTTCCTTCGGATTTCA 
RATTCCTTCCTGGCTTTGC 
*Caenorhabditis elegans clk-1*
FAGTGTGGCTGCTTATGCTCTCG
RGCTGAACCGACACCTGCAAGG
*Caenorhabditis elegans sod-3*
FCATTGTTTCAGCGCGACTTCGG
RTCCCCAGCCAGAGCCTTGAAC
*Caenorhabditis elegans gcs-1*
FTTCGGAATGGGGTGCTGTTGTC
RGAAGATTGGTGTGGCGGCAGAG
*Caenorhabditis elegans gst-4*
FGCTCAATGTGCCTTACGAGG
RGCAGTTTTTCCAGCGAGTCC
*Caenorhabditis elegans gst-7*
FTGACTTGAGCCTCCTCCCATGC
RTGACTTGAGCCTCCTCCCATGC
*Caenorhabditis elegans skn-1*
FATTCGTCGACGCGGAAAGAA 
RGGCTTTAATAAGGTTTCGACCGAG

### 4.9. Intracellular Localization of SKN-1::GFP 

Measurement of SKN-1 linked to green fluorescence protein (GFP) expression was performed following the protocol previously described by Xu et al. [22]. After the *skn-1(zu169)* mutants were treated with UVB, the worms were transferred into new NGM plates to culture for a time at 20 °C. The nematodes were then placed on 2% (*w*/*v*) agarose pads on glass slides and anesthetized with sodium azide (10 mM). The immobilized nematodes were detected under the laser scanning confocal microscope for observation. Three patterns were designated to define nuclear localization, namely cytosolic, intermediate, and nuclear. These indicated the invisible cellular localization, fluorescence that mostly concentrated on the head and tail, and fluorescent dots all over the body, respectively. The percentages of the three conditions were compared between the treatment groups and the control group for statistical analysis.

### 4.10. Statistical Analysis

Results from the abovementioned experiments were expressed as mean ± SD, and statistical analyses were performed using the GraphPad Prism 8.0.2 software (GraphPad software, San Diego, CA, USA). Differences between means were detected by ANOVA, and *p* < 0.05 was considered significant.

## 5. Conclusions

In this study, we first investigated the effect of different ethanol concentrations on the precipitation of UA in the *Cornus officinalis* crude extract. The results showed that almost all UA could be precipitated out at 40% ethanol concentration, while most other components were transferred to the supernatant. Then, COE was obtained via centrifugation followed by filtration and drying. Further research on the anti-photoaging activity and mechanisms of COE in the *C. elegans* model exposed to UVB indicated that COE could protect *C. elegans* from UVB-induced oxidative stress by eliminating excessive ROS, activating antioxidant enzymes, and affecting the expression of antioxidant-related genes in many signaling pathways. COE could stimulate the migration of SKN-1 into the nucleus. In addition, COE did not cause an extension in the lifespan of *skn-1(zu135)* mutants and inhibited the expression of *skn-1* downstream genes, indicating that SKN-1 is necessary for lifespan extension in *C. elegans* exposed to UVB. These results demonstrate that COE mainly ameliorates UVB-induced photoaging in *C. elegans* by activating the SKN-1/Nrf2 pathway. This study provides a reference for using COE as an active ingredient for skin protection from UVB irradiation. 

## Figures and Tables

**Table 1:** Effect of COE on the lifespan of *skn-1(zu135)* mutants under UVB stress. Values with different letters in column are significantly different (*p* < 0.05).

Group	Mean Lifespan (day)	% of Control
0 mg/mL	8.55 ± 0.46 a	100.00
1 mg/mL	8.23 ± 0.44 a	96.31
2 mg/mL	8.49 ± 0.74 a	99.38
4 mg/mL	8.49 ± 0.15 a	99.32

## Data Availability

Data are contained within the article and Supplementary Materials.

## Supplementary Materials

The following supporting information can be downloaded at: https://www.mdpi.com/article/10.3390/molecules29122718/s1. Figure S1: The chromatogram of samples, Table S1: The content of ursolic acid in the used samples.
