# Supplementary material for: Cornus officinalis Extract Enriched with Ursolic Acid Ameliorates UVB-Induced Photoaging in Caenorhabditis elegans
Source: Molecules. 2024 Jun 7;29(12):2718. doi: 10.3390/molecules29122718 (PMC11206114; doi:10.3390/molecules29122718)
Supplement: Supplementary file 1 [file molecules-29-02718-s001.zip › molecules-2960426-SI.pdf]

Supplementary Materials

A

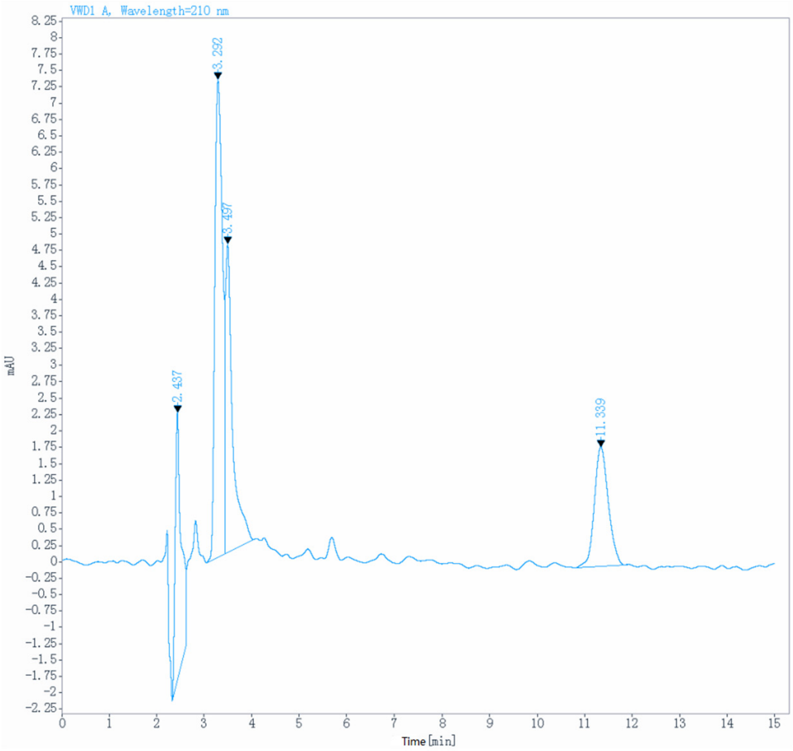

B

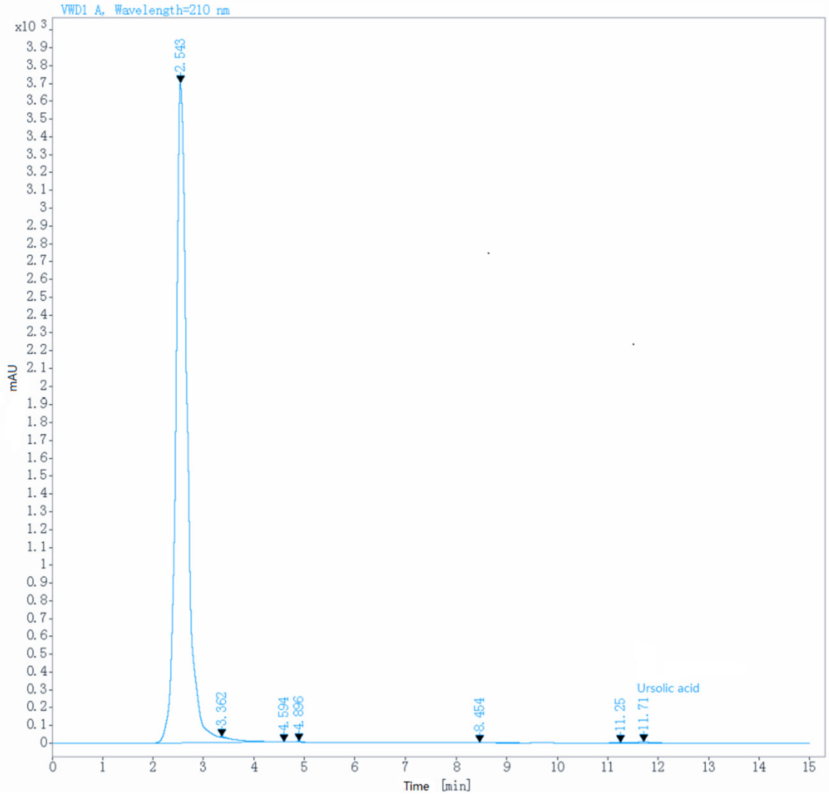

C

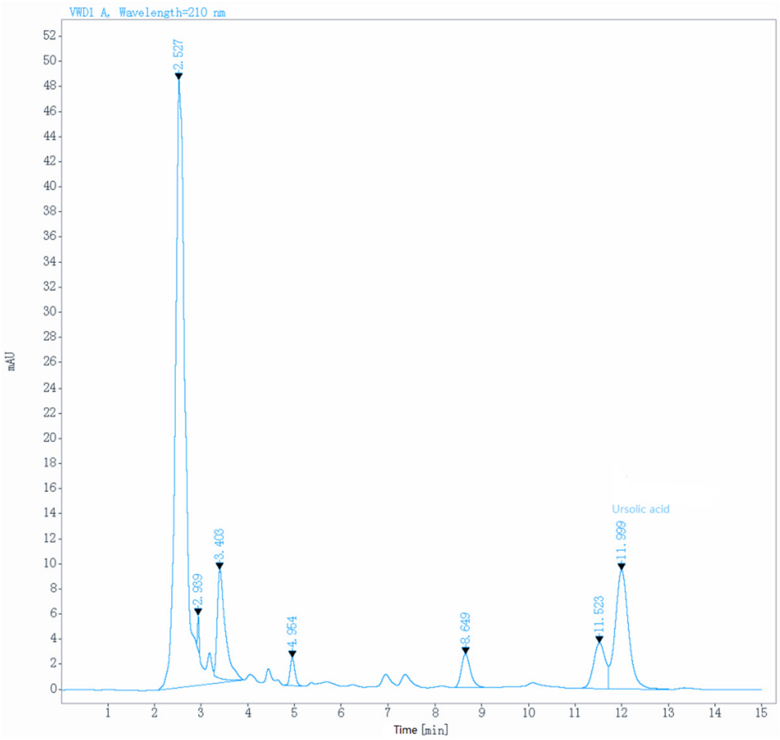

Figure S1. *The chromatogram of samples*. A. The HPLC of the standard solution of ursolic acid. B. The HPLC of the crude extract of *Cornus officinalis*. C. The HPLC of *Cornus officinalis* extract.

Table S1. The content of ursolic acid in the used samples.

**Samples**	**Content (μg/mg)**
The crude extract of *Cornus officinalis*	21.75±0.22
The *Cornus officinalis* extract	151.99±3.46
